# Analysis of Metal-Binding Features of the Wild Type and Two Domain-Truncated Mutant Variants of *Littorina littorea* Metallothionein Reveals Its Cd-Specific Character

**DOI:** 10.3390/ijms18071452

**Published:** 2017-07-06

**Authors:** Òscar Palacios, Elena Jiménez-Martí, Michael Niederwanger, Selene Gil-Moreno, Oliver Zerbe, Sílvia Atrian, Reinhard Dallinger, Mercè Capdevila

**Affiliations:** 1 Departament de Química, Facultat de Ciències, Universitat Autònoma de Barcelona, E-08193 Cerdanyola del Vallès, Spain; oscar.palacios@uab.cat (Ò.P.); selenebdn89@gmail.com (S.G.-M.); 2 Departament de Genètica, Facultat de Biologia, Universitat de Barcelona, Av. Diagonal 643, E-08028 Barcelona, Spain; ejimenezmarti@gmail.com (E.J.-M.); 3 Institute of Zoology and Center of Molecular Biosciences Innsbruck (CMBI), University of Innsbruck, Technikerstraße 25, A-6020 Innsbruck, Austria; michael.niederwanger@uibk.ac.at (M.N.); reinhard.dallinger@uibk.ac.at (R.D.); 4 Department of Chemistry, University of Zurich, 8057 Zurich, Switzerland; oliver.zerbe@chem.uzh.ch

**Keywords:** *Littorina littorea*, metallothionein, metal binding, tridominial MT

## Abstract

After the resolution of the 3D structure of the Cd_9_-aggregate of the *Littorina littorea* metallothionein (MT), we report here a detailed analysis of the metal binding capabilities of the wild type MT, LlwtMT, and of two truncated mutants lacking either the N-terminal domain, Lltr2MT, or both the N-terminal domain, plus four extra flanking residues (SSVF), Lltr1MT. The recombinant synthesis and in vitro studies of these three proteins revealed that LlwtMT forms unique M_9_-LlwtMT complexes with Zn(II) and Cd(II), while yielding a complex mixture of heteronuclear Zn,Cu-LlwtMT species with Cu(I). As expected, the truncated mutants gave rise to unique M_6_-LltrMT complexes and Zn,Cu-LltrMT mixtures of lower stoichiometry with respect to LlwtMT, with the SSVF fragment having an influence on their metal binding performance. Our results also revealed a major specificity, and therefore a better metal-coordinating performance of the three proteins for Cd(II) than for Zn(II), although the analysis of the Zn(II)/Cd(II) displacement reaction clearly demonstrates a lack of any type of cooperativity in Cd(II) binding. Contrarily, the analysis of their Cu(I) binding abilities revealed that every LlMT domain is prone to build Cu_4_-aggregates, the whole MT working by modules analogously to, as previously described, certain fungal MTs, like those of *C. neoformans* and *T. mesenterica*. It is concluded that the *Littorina littorea* MT is a Cd-specific protein that (beyond its extended binding capacity through an additional Cd-binding domain) confers to *Littorina littorea* a particular adaptive advantage in its changeable marine habitat.

## 1. Introduction

Metallothioneins (MTs) constitute a monophyletic superfamily [1] of highly heterogeneous proteins of, generally [2], a small size. They are present in almost all living organisms, and are able to coordinate a number of heavy-metal ions through the formation of metal-thiolate bonds via their highly abundant cysteine residues [3,4]. Their biological functions have been ascribed to the physiological regulation of Zn and Cu homeostasis and/or the detoxification of Cd and other toxic metals, although they also respond to different stress situations. In any case, the biological roles of MTs seem to be cell- and species-dependent, based on their reported heterogeneity. The latter has apparently evolved in a lineage-specific manner, according to the particular physiological requirements of the respective species [5]. Only rarely, however, was it so far shown how MT isoforms in a given species have adapted structural and metal-binding features in order to optimize metal-specific functions. One of the best-documented examples of this may be the metal-selective MT isoforms that evolved in certain terrestrial gastropods like the Roman snail (*Helix pomatia*) and some of its relatives [6,7]. On the other side, the plasticity required to perform a great multiplicity of functions could explain the extraordinary polymorphism reported for MTs, considering that in nearly all organisms analyzed so far, several coexisting MT isoforms have been found. After two decades devoted to the study of the metal binding abilities of a considerable number of MTs from diverse organisms [8], our group proposed a functional classification of MTs on the basis of their metal-binding preferences. This proposal, while acknowledging the classification of MTs according to sequence similarities within the taxonomic subfamilies [1,9], initially recognized two major groups [10]: Zn-thioneins (including both Zn(II) or Cd(II)-preferring MTs) and Cu-thioneins (i.e., Cu(I)-preferring MTs). Later, a step-wise gradation between genuine, Zn-(or divalent metal-ions)-thioneins and genuine Cu-thioneins was established [11]. In both of the extreme situations unique, well-folded, homometallic complexes were observed when bound to its cognate metal ion [8].

An ideal model system to study the evolutionary differentiation of polymorphic MTs, and the structure/function relationship in these metalloproteins, is the MT subfamily from the Mollusk class of Gastropoda (snails and slugs). Gastropods have existed as a distinct monophyletic clade for more than 500 million years [12]. Presently, they comprise a huge number of species (about 80,000) that have since successfully adapted to marine, freshwater, and terrestrial environments. As a reflection of this, gastropod MTs provide a fascinating example of how these proteins have evolved and diversified in such an ancient and diverging animal phylum. One of the most interesting aspects of gastropod MT evolution arises from the fact that species of the terrestrial helicid family possess metal-selective MT isoforms. The study of these peptides provided us with valuable data to recognize some distinct features that confer to them their specific Zn/Cd or Cu-thionein character. The paralogous MT proteins of these snails are, in fact, highly specialized for binding distinct metal ions while retaining high primary sequence similarities. Hence, the terrestrial snails *Helix pomatia* [6,7,13,14,15,16] and *Cantareus aspersus* [17,18,19], the best characterized snail MT systems so far, include three paralogous MT peptides with differentiated metal binding preferences: the Cd-specific (CdMT) and the Cu-specific (CuMT) isoforms, and an unspecific Cd/CuMT isoform that can be isolated as a mixed Cd,Cu-containing native complex, which have all been extensively studied by our group.

The marine common periwinkle *Littorina littorea* is also a member of Gastropoda that, in contrast to the terrestrial gastropods, has a unique MT. Interestingly it possesses a much longer sequence with 27 cysteine residues (Cys) instead of the 18 Cys commonly found in the other snails. *Littorina littorea* lives in a habitat (rocky sea shores) with rapidly changing environmental conditions due to tidal and microclimatic fluctuations, exposing snails to both marine and terrestrial conditions and alternating mineral and trace element availabilities, with an increasing risk of metal ion disbalances. Our research group has recently succeeded in determining the Nuclear Magnetic Resonance (NMR) structure of the Cd(II)-complexed form of *Littorina littorea* metallothionein, Cd_9_-LlMT. It appears that the protein possesses three individual domains, each of them forming an independent metal-chelating module that folds into a single, well-defined Cd_3_ cluster [20]. In comparison to MTs from other snail species that are only comprised by two domains, this novel three-domain MT is likely to confer to *Littorina littorea* an evolutionary advantage by structural adaptation to the higher risk of metal exposure in the marine tidal zone, through a simple domain duplication event. Overall, the MT of *Littorina littorea* seems to have adapted to stressful environmental conditions in a twofold manner: first, by increasing the metal binding specificity of the protein towards Cd(II) (present study); and second, by addition of an extra metal binding domain so as to increase the metal/protein stoichiometry from six to nine Cd(II) ions [20].

Hence, in the present study we explored in depth the Zn(II)-, Cd(II)- and Cu(I)-binding capabilities of the wild type *Littorina littorea* metallothionein, from now on referred to as LlwtMT, and of two designed mutants (Lltr1MT, Lltr2MT), comprising only two of the putative metal binding domains, with or without four N-terminal flanking residues.

## 2. Results and Discussion

### 2.1. Characterization of the Metallothionein (MT) System of Littorina littorea

**Figure 1:**
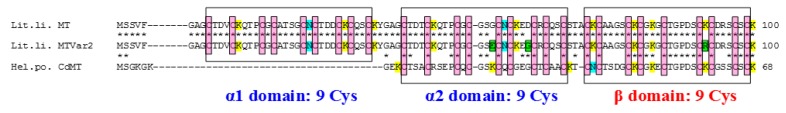
Alignment of *Littorina littorea* Metallothionein (MT) (Lit.li. MT, GenBank Acc. No. AAK56498), with its allelic variant (Lit.li. Var2, GenBank Acc. No. KY963497), and with the paradigmatic Cd-specific MT isoform of the terrestrial snail, *Helix pomatia* (Hel.po. CdMT, GenBank Acc. No. AAK84863.1). Throughout, conserved cysteine (Cys) positions in the peptide chains are highlighted by a **pink** box. In the sequence of the allelic Lit.li. MTVar2, the amino acid positions exchanged with respect to the wildtype Lit.li. MT sequences are shown in **green**. Also highlighted in **yellow** are the Lysine (K) residues whose preponderance over Aspargine (N) (highlighted in **blue**) in the sequences is supposed to confer to the respective peptides a high Cd(II) binding preference [19]. The transparent boxes indicate the supposed three-domain structure of the three MT proteins, which has been experimentally verified by solving the structure of *Littorina littorea* MT [20]. According to this, the *Helix pomatia* CdMT consists of two modular domains (one α2 and one β domain), whereas the two MT variants of *Littorina littorea* comprise three modular domains (two α domains, i.e., α1 and α2, and one β domain). In each of the above-shown proteins, every domain includes 9 Cys residues that provide 9 sulfur atoms for the binding of 3 divalent metal ions (such as Cd(II)). Identical positions between adjacent sequences are indicated by stars. The number of residues in the respective peptide chains is specified near their C-terminal end.

The first primary structure knowledge of an MT from *Littorina littorea* goes back to studies of English and Storey [21], who recognized the important role of this MT in response to environmental stressors such as freezing and anoxia, to which the snail may intermittently be exposed in its tidal habitat. A screening of transcriptome from the midgut gland of Cd-exposed *Littorina littorea* for possible additional MT isoforms of this species by our team was negative, while the originally proposed sequence could be retrieved and confirmed via Polymerase Chain Reaction (PCR). Hence, it is actually assumed that the formerly identified MT of *Littorina littorea* [21] may be the only isoform from this species. Its sequence was therefore used in the present study. Interestingly, however, we found some variability of this MT in terms of an allelic variant that differs from the wild type MT in a few amino acid positions (Figure 1). While these slight primary structure differences may not significantly impact the overall metal binding behavior between wild type and allelic variants, their sequence composition with 9 Cys residues (i.e., 9 metal binding sulfur atoms) for each putative cluster suggest the presence of three individual domains, each of them carrying one metal cluster with a stoichiometric ratio of 9 Cys residues versus 3 divalent or 6 monovalent metal ions (Figure 1). The three-domain structure of this MT was in fact recently confirmed by our group using solution NMR [20]. In contrast, the metal binding features of the *Littorina littorea* MT remained still uncharacterized, and are now presented in this study.

### 2.2. The LlwtMT, Lltr1MT and Lltr2MT Recombinant Polypeptides

**Figure 2:**
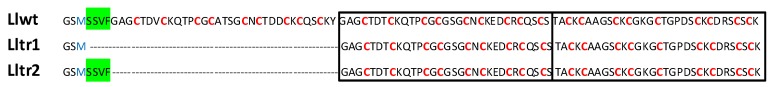
Sequences of the recombinant proteins studied in this work: the constructs Lltr1MT (GeneBank Accession No. MF326202) and Lltr2MT (GeneBank Accession No. MF326203) are aligned with the LlwtMT (UniProt #Q962G0) wild-type form. Cysteine residues are written in **red**, Methionine residues in **blue**, and the SSVF N-terminal flanking amino acids are highlighted in **green**.

**Figure 3:**
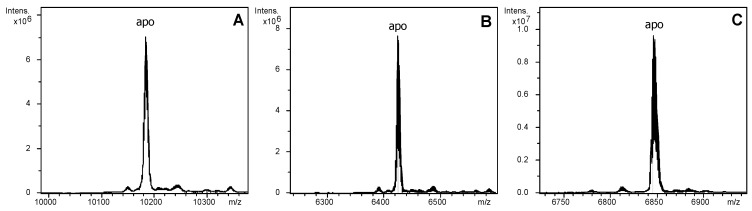
Deconvoluted Electrospray Ionization Mass Spectrometry (ESI-MS) spectra of the recombinant preparations of (**A**) LlwtMT; (**B**) Lltr1MT; and (**C**) Lltr2MT, purified from bacterial cultures grown under Zn-supplementation and analyzed at acid pH (2.4).

In order to understand the possible impact of the number and length of domains on the metal binding properties of the *Littorina littorea* MT, the wild type MT was compared with two domain-truncated mutants lacking one of the two α-domains. As shown in our previous publication [20], the increase in Cd loading capacity of the *Littorina littorea* MT has been achieved by duplication of the N-terminal α-domain (resulting in an MT with two N-terminal α-domains and one C-terminal β-domain). Our hypothesis was that the evolutionary duplication of the α-domain (and resulting addition of one more domain) should confer to the MT an increased loading capacity without grossly impairing its metal specificity features and metal binding behavior. Therefore, the two MT mutants were designed in order to contain only two metal binding domains (one single α and one single β domain), like the most common snail MTs (Figure 2). One of them (Lltr1MT) lacks the N-terminal metal binding domain from amino acid positions 2 to 37. In the Lltr2MT mutant residues 6 to 37 were removed, so that the truncated protein presents the two C-terminal metal binding domains and a small stretch of four additional amino acids (-SSVF-) at its N-terminus. DNA sequencing of the cDNA of the three proteins confirmed their sequence. Expression in *E. coli* cultured in Zn-enriched media and purification rendered the corresponding recombinant Zn-LlwtMT, Zn-Lltr1MT, and Zn-Lltr2MT samples. These, once acidified, yielded the corresponding apo-forms, with respective molecular masses of 10,183, 6425, and 6846 Da, in accordance with the respective theoretical values of 10,183.59, 6426.35, and 6846.82 Da (Figure 2 and Figure 3, Table 1), respectively.

### 2.3. Zn(II) and Cd(II) Binding Capabilities of LlwtMT, Lltr1MT, and Lltr2MT

**Figure 4:**
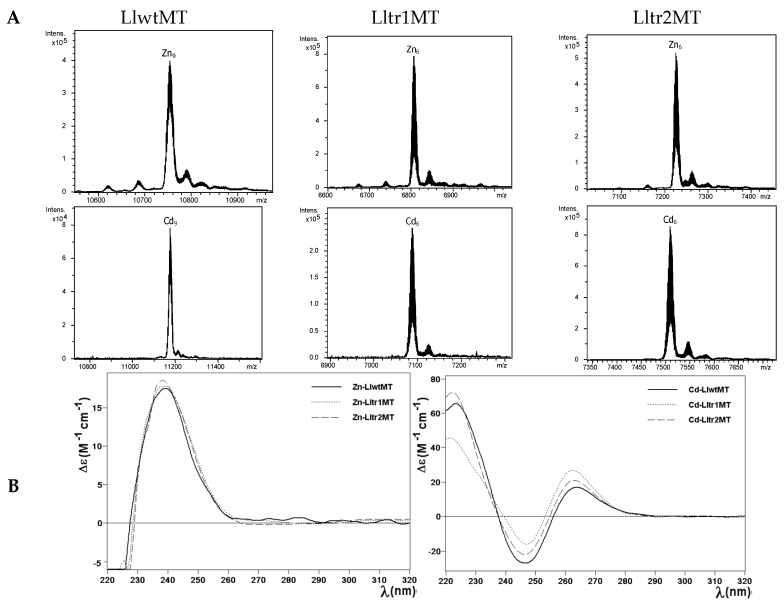
Analysis of the Zn- and Cd-LlwtMT, -Lltr1MT, and -Lltr2MT complexes. (**A**) Deconvoluted ESI-MS spectra of the recombinant preparations of LlwtMT, Lltr1MT, and Lltr2MT, purified from Zn- and Cd-supplemented cultures, analyzed at neutral pH (7.0); (**B**) CD spectra of the corresponding Zn- and Cd-LlMT recombinant preparations.

**Figure 5:**
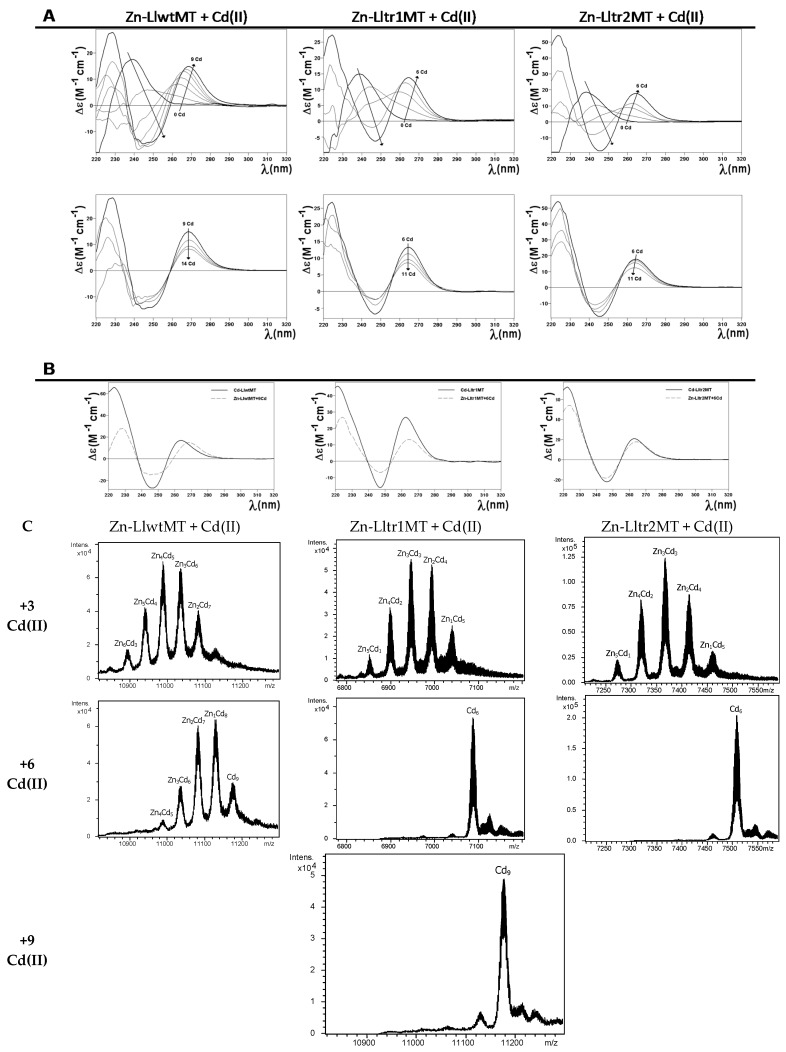
Zn(II)/Cd(II) replacement reaction on the Zn_9_-LlwtMT, Zn_6_-Lltr1MT, and Zn_6_-Lltr2MT complexes. (**A**) CD spectra of a 10 μM solution of the Zn-LlMT samples, titrated with CdCl_2_ at neutral pH up to 14 (LlwtMT) or 11 (Lltr1MT and Lltr2MT) Cd(II) equivalents; (**B**) Comparison of the CD spectra of the three recombinant productions of Cd-LlMT with those obtained at the end of the in vitro metal displacement reactions; (**C**) Deconvoluted ESI-MS spectra recorded after the addition of 3, 6, and 9 equivalents of CdCl_2_ to the recombinant Zn-LlMT preparations.

The recombinant synthesis of LlwtMT in M^II^-supplemented (M^II^ = Zn(II) or Cd(II)) *Escherichia coli* cultures yielded almost unique peaks corresponding to M^II^_9_-complexes, while those of the truncated mutants, Lltr1MT and Lltr2MT, gave rise to M^II^_6_-complexes for both peptides, as identified in the respective ESI-MS analyses at neutral pH (Figure 4A and Table 1). The only minor accompanying peaks were attributed to frequently observed NH_4_^+^ adducts. These results are in good concordance with those obtained previously for LlwtMT synthesized under equivalent conditions [20], and nicely match with those expected after removing one of the structural domains, and therefore reduce the initial 27 to 18 Cys coordinating amino acids. Analysis of the Circular dichroism (CD) spectra of the Zn- and Cd-preparations of LlwtMT and of those of the Lltr1MT and Lltr2MT truncated mutants (Figure 4B) confirmed that, irrespective of their metal loading (9 M^II^ metal cations but 6 M^II^ in the truncated forms), (1) the three Zn-loaded proteins showed equivalent folds, and (2) the three Cd-loaded proteins also showed equivalent folds. However (3), the Zn(II) and the Cd(II) complexes of these three proteins did not show the same level of structuration; in fact, peptides loaded with Cd(II) were more well-structured in solution, as can be deduced from the *exciton coupling* signal centered at ca. 255 nm, which is a characteristic wavelength of the Cd-thiolate chromophores (Figure 4B, right hand). In contrast, the corresponding Zn-loaded complexes gave rise to the typical Zn(SCys)_4_ absorption, with a Gaussian band centered at ca. 240 nm (Figure 4B, left hand), representative of a lower degree of compactness and a less defined 3D structure in solution. Furthermore, the Zn(II)/Cd(II) displacement reaction in Zn_9_-LlwtMT, Zn_6_-Lltr1MT, and Zn_6_-Lltr2MT proceeded gradually from the Zn_x_-LlMT complexes to the respective Cd_x_-LlMT species (x = 9 or 6), giving rise to all the Zn_a_Cd_b_-LlMT (a + b = x) intermediate species in the transitional steps (Figure 5). The CD spectra recorded at progressive stages of the reaction revealed identical profiles (cf. Figure 5A) for the three polypeptides. The observed changes basically consisted in the transition of the initial Gaussian band at ca. 240 nm, characteristic of the Zn-complexes, into the exciton coupling signal centered at ca. 250 nm, typical of Cd-complexes. This suggests that the Zn(II)/Cd(II) substitution proceeds in an almost parallel way for the wild-type LlwtMT and the two mutant forms. The CD spectra of the Zn/Cd replacement steps also suggest non-cooperative replacement (Figure 5A), which is supported by the ESI-MS data (Figure 5B).

After the respective additions of 9 and 6 Cd(II) equivalents to the recombinant Zn-LlMT preparations, the respective peptides exclusively yielded the expected Cd_9_-LlwtMT, Cd_6_-Lltr1MT, and Cd_6_-Lltr2MT complexes (Figure 5C), in agreement with the results obtained in the in vivo recombinant preparations and the reported NMR results [20]. These in vitro-generated complexes show CD fingerprints almost equivalent to those of the in vivo-synthesized species (Figure 5B).

Therefore, it can be concluded that the removal of one of the structural domains of the LlwtMT protein does obviously affect the overall metal content of the final aggregates but likely not the structure of the individual domains.

### 2.4. Cu(I) Binding Capabilities of LlwtMT, Lltr1MT, and Lltr2MT

**Figure 6:**
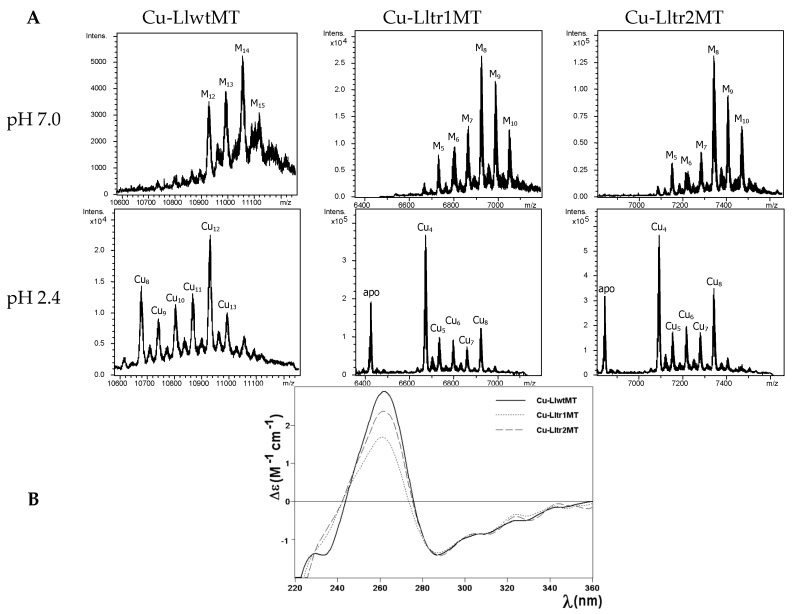
Analysis of the Cu-LlwtMT, Cu-Lltr1MT, and Cu-Lltr2MT complexes. (**A**) Deconvoluted ESI-MS spectra of the recombinant preparations of LlwtMT, Lltr1MT, and Lltr2MT, purified from Cu-supplemented cultures, analyzed at neutral pH (7.0) and acid pH (2.4); (**B**) CD spectra of the three recombinant Cu-LlMT preparations.

Due to the known influence of oxygenation on the amount of internal copper in the cultured bacteria [22], and following our established methodology [8], we performed two types of Cu-supplemented productions: one under standard and one under low aeration conditions. The synthesis of the three polypeptides at regular oxygenation conditions yielded preparations that allowed their analysis by ESI-MS and CD, and facilitated the comparison of all their features. Unfortunately, several efforts to purify LlwtMT, Lltr1MT, and Lltr2MT from *E. coli* cultures grown under low oxygen conditions failed. This fact, together with the results described below (that clearly illustrate the degree of heterogeneity of the Cu-LlMT recombinant samples (Figure 6A)) and the results obtained in the M^II^ binding studies (see above), already suggest a low specificity of these three proteins for Cu(I). At this point we would like to remind the reader of data obtained in some of our previous Cu(I) binding studies, performed with other snail MTs [15], where other Cu-MT preparations also exhibited a high degree of complexity when synthesized in the presence of a non-cognate metal ion.

The first noticeable observation was that the composition of the Cu-LlwtMT and Cu-LltrMT purified samples was significantly different (obviously, this was expected, owing to their different number of coordinating residues), apart from the manifold of different species and the heteronuclear nature (i.e., Cu,Zn-species) of the complexes found in the three Cu-LlMT samples. For example, the ESI-MS spectra of the Cu-LlwtMT sample recorded at neutral pH exhibited one major peak, corresponding to the M_14_-LlwtMT complexes; two less abundant M_13_- and M_12_-LlwtMT, and one very minor M_15_-LlwtMT species (Figure 6A, Table 2). Due to the similarity of their atomic masses M can be either Zn(II) or Cu(I). The same preparation was analyzed at acidic pH, as this allows the release of all bound Zn(II) but not of Cu(I) [23,24]. At pH 2.4 the major peak corresponded to homonuclear Cu_12_-LlwtMT, and a smaller peak for Cu_8_-LlwtMT as well as minor peaks for Cu_9_-, Cu_10_-, Cu_11_-, Cu_13_- and Cu_14_-LlwtMT (Figure 6A, Table 2). Analogously, the Cu-Lltr1MT and Cu-Lltr2MT preparations resulted in major M_8_- with also abundant M_9_-complexes, while the M_10_-, M_7_-, and M_6_-species appeared as minor MS peaks. At acidic pH these samples rendered major Cu_4_-complexes, less abundant Cu_8_-species, and minor Cu_5_-, Cu_6_- and Cu_7_-species, together with a relatively intense peak (corresponding to the truncated apo-proteins, a peak that was not observed for wild type Cu-LlwtMT). In any case, the ESI-MS data at acidic pH clearly indicated that the Cu_4_-, Cu_8_-, and Cu_12_-aggregates were the most favored ones in these LlMTs, thus suggesting an optimal occupancy of 4 Cu(I) ions per structural domain. The ICP-AES results obtained for the purified Cu-LlwtMT, -Lltr1MT, and -Lltr2MT samples (4.0 Zn:13.3 Cu; 3.5 Zn:5.8 Cu; and 3.2 Zn:6.5 Cu, respectively, for LlwtMT, Lltr1MT, and Lltr2MT, see Table 2) revealed that the deletion of one out of the three structural domains of LlwtMT has drastically reduced the copper content to one half, but practically maintained that of Zn. The most straightforward explanation for the overall results may be that according to our previous observations [19], the small number of asparagine (N) residues (two in the LlwtMT, see Figure 1 and Figure 2) was reduced by half in the two truncated LlMTs (one asparagine residue left in each, Lltr1MT, and Lltr2MT), hence decreasing the already low Cu(I) binding character of the LlwtMT even further in the two truncated Lltr1MT and Lltr2MT. Another explanation would be that Cu-LlwtMT contains Cu_12_- and Cu_8_-aggregates, which also enclose some Zn(II) ions to finally render the observed M_12_-, M_13_-, and M_14_-LlwtMT species. In contrast, the truncated mutants present Cu_4_-aggregates that, however, still contain Zn(II) to yield the observed M_8_- and M_9_-complexes. The fact that these preparations also showed a significantly intense peak corresponding to their apo-peptides, at pH 2.4, suggests that some of the complexes are only loaded with Zn(II). Finally, the relative higher intensity of Cu_8_-Lltr2MT, in comparison to that of Cu_8_-Lltr1, seems to indicate that the SSVF fragment perhaps has a role in the stabilization of the aforementioned Cu_4_-aggregates.

Interestingly, despite the diversity of species formed by the three LlMT proteins when coordinating Cu(I), they all gave rise to comparable CD spectra, both in shape and intensity (Figure 6B). Their envelopes display the typical fingerprints of the Cu-MT complexes with absorption maxima at ca. 260 nm and minima at ca. 280 nm.

**Figure 7:**
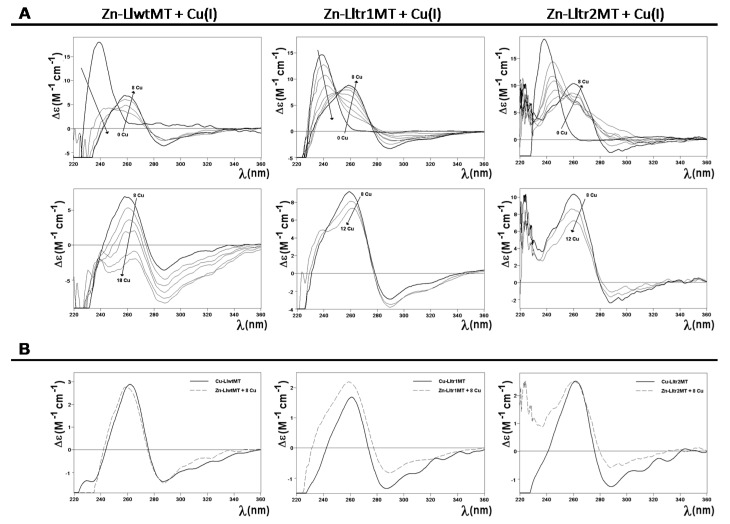
Zn(II)->Cu(I) replacement reaction on the Zn_9_-LlwtMT, Zn_6_-Lltr1MT, and Zn_6_-Lltr2MT complexes. (**A**) CD spectra of a 10 μM solution of the Zn-LlMT samples, titrated with a Cu(I) solution at neutral pH; (**B**) Comparison of the CD spectra (normalized) of the three recombinant productions of Cu-LlMT with those obtained from the in vitro metal displacement reactions.

**Figure 8:**
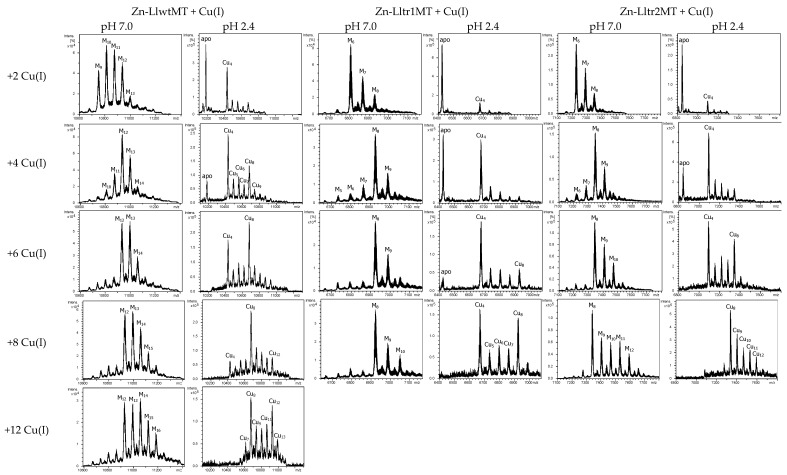
Zn(II)->Cu(I) replacement reaction on the Zn_9_-LlwtMT, Zn_6_-Lltr1MT, and Zn_6_-Lltr2MT complexes. Deconvoluted ESI-MS recorded, at pH 7.0 and 2.4, after the addition of 2, 4, 6, 8, and 12 equivalents of Cu(I) to the recombinant Zn-LlMT preparations.

To further explore the Cu(I)-binding capabilities of these three peptides, their Zn-loaded forms were treated with increasing amounts of a Cu(I) solution. The distinct stages reached during the titrations were analyzed by CD and ESI-MS, and compared with the results obtained from the recombinant Cu-LlMT preparations (Figure 7 and Figure 8). As expected, the Zn(II)->Cu(I) displacement reaction in Zn_9_-LlwtMT, Zn_6_-Lltr1MT, and Zn_6_-Lltr2MT gave rise, at pH 7, to a complex mixture of heterometallic M_x_-LlMT species (Figure 8). The 240 nm Gaussian band in the CD spectra, characteristic of the Zn-loaded form, decreased in intensity in all three peptides and shifted towards the red with the incoming Cu(I). After the addition of 8 equivalents of Cu(I), the typical CD envelop characteristic of Cu-loaded MTs (with absorptions at ca. 260, 290, and 320 nm (cf. Figure 7A)) was observed. At this stage both, the CD fingerprints (Figure 7B) and the metal composition (Figure 8), as deduced from the MS data, were quite close (but not exactly equal) to those observed in the recombinant synthesis of the three proteins (Figure 6). Further addition of Cu(I) beyond 8 Cu(I) equivalents resulted in all three samples in a decrease of the CD signal (Figure 7B). The Zn(II)->Cu(I) substitutions in vitro proceeded in an almost parallel way for the wild-type LlwtMT and the two truncated LltrMT forms, showing CD fingerprints almost equivalent to those of the in vivo synthesized species (Figure 7B).

The ESI-MS data at acidic pH, however, revealed a distinctly different behavior for each peptide. First of all, it is worthwhile to highlight that the spectrometric measurements at acidic pH revealed that the three peptides formed copper aggregates on the basis of Cu_4_-clusters, in closer resemblance to the behavior observed for the two MTs (CnMT1 and CnMT2) of the pathogenic fungus *Cryptococcus neoformans*. It was recently suggested that these MTs are built from a modular structure of Cu_5_ clusters [24], in response to a highly selective pressure to chelate copper. Hence in our present study, the addition of a few Cu(I) equivalents at the beginning of the three experiments already gave rise to the appearance of Cu_4_-LlMT clusters, which remained very abundant, while Cu_8_-LlMT clusters gained in importance when further Cu(I) was added. After the addition of the fourth Cu(I) equivalent, at which a significant amount of the apo protein was still present, M-LlwtMT species already contained a significant fraction of the Cu_8_ cluster. This was not the case for both truncated mutants, for which the apo-protein and Cu_4_ presented the major species. Interestingly for the six Cu(I) equivalents added, Cu_8_ was already the major species for LlwtMT. Oppositely, Lltr1Mt maintained a small proportion of the apo-protein and showed an increased amount of Cu_8_, while Lltr2 had no apo-protein and a higher amount of Cu_8,_ in regards to that of Cu_4_. With 8 Cu(I) equivalents added, Cu_8_ was the major species for LlwtMT and Lltr2MT but not for Lltr1MT, for which a significant fraction was present as the Cu_4_ aggregate. Addition of excess Cu(I) did not induce further changes in the metal content of the truncated mutants, which indicates that Lltr2MT, but not Lltr1MT, can easily reach a state in which both domains are loaded with 4 Cu(I). We suspect that the additional presence of the SSVF amino acid motif at the N-terminus of Lltr2MT is important for that feature. On the other hand, and as expected, LlwtMT can bind more Cu(I) equivalents so that its third domain becomes loaded with further 4 Cu(I) ions, resulting in a Cu_12_ cluster as the fully saturated species.

## 3. Materials and Methods

### 3.1. Confirmation of the MT System of Littorina littorea

Individuals of *Littorina littorea* (20–30 mm high) collected in Scrabster (Scotland) were obtained through a commercial dealer in Bilbao (Arrainko SL, Mercabilbao, Bilbao, Spain). After an acclimation period of two weeks, twenty snails were dissected at the Research Centre for Experimental Marine Biology and Biotechnology (University of the Basque Country) in Plentzia (Bizkaia, Basque Country, Spain). Midgut gland aliquots (~10 µg) were separated and stored in RNA-later for subsequent RNA isolation at the Institute of Zoology of the University of Innsbruck (Innsbruck, Austria). RNA isolation of homogenized (Precellys, Bertin Instruments, Montigny-le-Bretonneux, France) hepatopancreatic tissue was performed using the RNeasy^®^Plant Mini Kit (Qiagen, Hilden, Germany), applying on-column DNase 1 digestion (Qiagen). RNA was quantified using RiboGreen^®^RNA Quantification Kit from Molecular Probes (Invitrogen, Karlsruhe, Germany). The RNA sample of one individual was sent to Duke University (Durham, NC, USA) for Illumina HiSeq Sequencing, in order to screen the transcriptome for the presence of additional MT isoforms (which, however were not present). The allelic variant 2 was discovered in a screen of twenty individuals of *Littorina littorea*.

cDNA was synthesized from 450 ng of total RNA with the Superscript^®^ IV Reverse Transcriptase synthesis kit (Invitrogen, Life Technologies, Waltham, MA, USA) on a 20 µL scale. The primary structure of the *Littorina littorea* MT (GenBank Acc.Nr. AAK56498) and the allelic variant 2 (GenBank Acc.Nr. KY963497) were confirmed by PCR. To this aim, the Titanium^®^ Taq PCR Kit (Clontech, Mountain View, CA, USA) was used with the following primers: 5′UTR primer, 5′-CTGACGAGTGAACTGTTTTT-3′; 3′UTR primer, and 5′-GATGGGGAATGAGAAAATG-3′.

### 3.2. Construction and Cloning of the cDNAs Encoding the LlwtMT, Lltr1MT, and Lltr2MT Proteins

Two LltrMT truncation mutants (LltrMT1 and LltrMT2) were designed to lack the first metal binding domain completely. In Lltr1, the N-terminal domain (including all amino acid positions from 2 to 37) were deleted, while in Lltr2 the N-terminal domain was truncated too, but the N-terminal amino acid positions from 1 to 5 were maintained (Figure 2).

For the construction of the pGEX plasmids containing the wild type sequence of *Littorina littorea* or the mutants, the cDNAs encoding for these sequences were designed on the basis of the flanking regions in wild-type LlwtMT cDNA, and to encode the truncated sequences in the corresponding Lltr1MT and Lltr2MT cDNAs, taking out nucleotides 4 to 111 and 16 to 111, respectively. Additionally, the restriction sites for *BamHI* and *XhoI* were added to the 5′ and 3′ ends, respectively, for cloning purposes (Supplementary Materials, Figure S1). The LlwtMT, Lltr1MT, and Lltr2MT coding sequences designed this way were purchased as synthetic DNAs IDT & Conda Labs (Madrid, Spain). After PCR amplification (35 cycles: 95 °C 30 s, 50 °C 30 s, and 72 °C 30 s, using Expand High Fidelity (Roche Diagnostics S.L:, San Cugat del Vallès, Spain) thermostable DNA polymerase) of the synthetic cDNAs using the flanking primers 5′-TTTATTGGATCCATGAGCTC-3′ (LlwtMT forward); 5′-TTTCTCGAGTCATTTGCATG-3′ (LlwtMT reverse); 5′-TTTATTGGATCCGGTAAGGG-3′ (Lltr1MT forward); 5′-TTTCTCGAGTTACTTACAGG-3′ (Lltr1MT reverse); 5′-TTTATTGGATCCGGCAAAGGG-3′ (Lltr2MT forward); and 5′-TTTCTCGAGTTATTTGCAAG-3′ (Lltr2MT reverse), they were subsequently digested with *BamHI* and *XhoI* restriction enzymes, and the resulting products were ligated in-frame (DNA ligation kit, Takara Bio, Kusatsu, Shiga, Japan) in the pGEX-4T-1 (Amersham-GE Healthcare Europe, Cerdanyola del Valles, Spain) *E. coli* expression vector, which yields GST-fusion proteins. DNA sequencing allowed confirming of all the DNA constructs (ABIPRISM 310, Applied Biosystems, Foster City, CA, USA) using the BigDye Terminator. The *E. coli* MachI strain was used for cloning and sequencing. The expression plasmids were then transformed into the protease-deficient *E. coli* strain BL21 (*fhuA2 [lon] ompT gal [dcm] ΔhsdS*) for protein synthesis.

### 3.3. Synthesis and Purification of the Recombinant Zn-, Cd-, and Cu-Complexes of LlwtMT and the Lltr1MT and Lltr2MT Mutants

Purification of all the metal-MT complexes of the LlwtMT, Lltr1MT, and Lltr2MT proteins was carried out, as reported previously [25], to ensure fully comparable results. Hence, the GST-LlMT fusions were produced in 5-L cultures (Luria Bertani medium) of transformed *E. coli* BL21 bacteria. Induction of gene expression was achieved with 100 μM (final concentration) of isopropyl β-d-thiogalactopyranoside (IPTG). After 30 min of induction, 300 μM ZnCl_2_, 300 μM CdCl_2_, or 500 μM CuSO_4_ (final concentrations) were supplemented to the cultures, which grew for a further 2.5 h, for the synthesis of the respective metal complexes. Owing to the well-known fact that culture aeration determines the amount of available intracellular copper [22], the Cu-cultures were grown both under normal (1-L medium in a 2-L Erlenmeyer flask, at 250 rpm) and low-oxygen conditions (1.5-L medium in a 2-L Erlenmeyer flask at 150 rpm).

Harvesting and centrifugation of the grown cells produced a cell mass that, resuspended in ice-cold PBS (1.4 M NaCl, 27 mM KCl, 101 mM Na_2_HPO_4_, 18 mM KH_2_PO_4_) with 0.5% *v*/*v* β-mercaptoethanol, was disrupted by sonication (20 s pulses for 5 min). All solutions used were oxygen-purged by saturating them with pure-grade argon to prevent metal-MT oxidation. The suspension was centrifuged at 12,000× *g* for 30 min, and the incubation of the resulting supernatant (gentle agitation for 60 min at room temperature) with Glutathione-Sepharone 4B (GE Healthcare) allowed batch affinity purification of the GST-LlMT species. The MT portion was recovered after thrombin cleavage (10 μ per mg of fusion protein at 17 °C over-night). The solution containing the cleaved metal-MT complexes was concentrated by Centriprep Microcon 3 (Amicon, cut-off of 3 kDa, Merck-Millipore, Darmstadt, Germany) centrifugation. The final metal complexes were purified through FPLC size-exclusion chromatography in a Superdex75 column (GE Healthcare), equilibrated with 50 mM Tris-HCl (pH 7.0), and run at 0.8 mL·min^−1^. Absorbances at 254 and 280 nm signaled the fractions to be collected and analyzed for protein content.

### 3.4. Zn(II)/Cd(II) and Zn(II)/Cu(I) Replacement Reactions in the Zn(II)-LlMT Proteins

Metal displacement reactions on the recombinant Zn-LlMT preparations allowed formation of the alleged Cd- or Cu-LlMT “in vitro complexes”. As described elsewhere [25,26], the respective additions of several molar equivalents of Cd^2+^ or Cu^+^ ions from standard solutions were performed at constant pH 7.0 without the addition of any extra buffers, and under argon atmosphere.

### 3.5. Spectroscopic Analyses (ICP-AES, UV-Vis and CD) of the Metal Complexes Formed by the Llmt Proteins

Inductively Coupled Plasma Atomic Emission Spectroscopy (ICP-AES) in a Polyscan 61E (Thermo Jarrel Ash, Franklin, MA, USA) spectrometer allowed determination of the sulfur and metal content of all the metal-MT samples, by measuring S at 182.040 nm, Zn at 213.856 nm, and Cu at 324.803 nm. The protein concentration was determined both by conventional treatment [27] and by incubation in 1 M HNO_3_ at 65 °C for 10 min, before measurements to avoid possible traces of labile sulfide anions [28], by assuming that all S atoms were provided by the MT proteins. Circular dichroism measurements were performed at 25 °C in a Jasco spectropolarimeter (Model J-715, JASCO, Groß-Umstadt, Germany) interfaced to a computer (J700 software, JASCO, Groß-Umstadt, Germany) by using Peltier PTC-351S equipment (TE Technology, Traverse City, MI, USA). An HP-8453 Diode array UV-vis spectrophotometer (GIM, Ramsey, MN, USA) was used for the electronic absorption measurements. In all cases, 1-cm capped quartz cuvettes were employed for spectra recording, and the dilution effects were corrected and processed using the GRAMS 32 software (Thermo Fisher Scientific, Waltham, MA, USA).

### 3.6. Electrospray Ionization Time-of-Flight Mass Spectrometry (ESI-TOF MS) of the Metal Complexes Obtained from the LlMT Proteins

Mass determinations by Electrospray ionization time-of-flight mass spectrometry (ESI-TOF MS) were carried out in a Micro TOF-Q instrument (Bruker Daltonics, Bremen, Germany) interfaced with a Series 1200 HPLC Agilent pump and equipped with an autosampler, all of which were controlled by the Compass Software. The ESI-L Low Concentration Tuning Mix (Agilent Technologies, Santa Clara, CA, USA) was used for calibration.

A 5:95 mixture of acetonitrile:ammonium acetate (15 mM) was the carrier buffer for measurements at pH 7.0, while the measurements at acidic pH (2.4) were carried out using a 5:95 acetonitrile:formic acid solution, which causes the release of Zn(II) but keeps Cu(I) bound to the peptides. Experimental mass values were calculated as described previously [29], and the error associated with the measurements was always smaller than 0.1%.

## 4. Conclusions

In summary, we have explored and analyzed the metal binding capabilities of wild type *Littorina littorea* MT (LlwtMT) and two truncated mutants that either lack the N-term domain (Lltr2MT), or both the N-term domain plus a short fragment (residues SSVF) at the N-terminus of its sequence (Lltr1MT).

The LlwtMT protein, with 27 Cys distributed equally in 3 structural domains [20], rendered unique M_9_-LlwtMT species—both in vivo and in vitro—when bound to divalent metal ions (M = Zn(II) or Cd(II)), while affording a complex mixture of heteronuclear Zn,Cu-LlwtMT species if Cu(I) was introduced. This already excludes that LlwtMT behaves like a Cu-thionein. Both truncated mutants matched the expectation that metal binding is simply scaled when removing a single domain from the full-length protein, e.g., obtaining unique M_6_-LltrMT complexes with divalent metal ions and a complex mixture of heteronuclear Zn,Cu-LltrMT species of lower stoichiometry, compared to the wild type protein (M_8_ instead on M_14_, M = Zn + Cu).

The characterization of recombinant preparations of these three proteins revealed increased specificity for Cd(II) over Zn(II), as clearly revealed by the CD fingerprints of their in vivo and in vitro preparations, that displayed Gaussian bands for the Zn-loaded complexes (Zn_9_-LlwtMT and Zn_6_-LltrMT) but clear *exciton couplings* for the Cd-loaded complexes (Cd_9_-LlwtMT and Cd_6_-LltrMT) at the corresponding wavelengths (ca. 240 nm for Zn(II) complexes, and ca. 250 for Cd(II) complexes). Despite the observed better folding of the proteins with Cd(II) compared to those with Zn(II), the metal displacement reaction of Zn(II) by Cd(II) did not reveal any type of cooperativity upon Cd(II) binding. The spectrometric data clearly revealed that the incoming Cd(II) ions gradually displaced the bound Zn(II) ions, initially yielding all the possible Zn_X_,Cd_y_-LlMT (x + y = 9 for LlwtMT, and x + y = 6 for LltrMT) species, until only Cd(II) was present in the final homonuclear Cd-LlMT complexes. Contrarily, the analysis of the Cu(I) binding capabilities of these proteins has revealed that the three domains are prone to form Cu_4_-aggregates in separate modules, as previously described for other MTs (like those of *Cryptococcus neoformans* [24] and *Tremella mesenterica* [2], which have been reported to have been built by tandem amplification of a basic unit).

Overall, the MT of *Littorina littorea* must be considered as a particularly efficient Cd-specific MT. It confers to the snail an evolutionary advantage in a twofold manner, because: (1) it is prone to preferentially bind Cd^2+^; and (2) as shown by us previously [20], its Cd binding capacity has been increased during evolution through the addition of a third metal binding domain.

## Figures and Tables

**Table 1:** Analytical characterization of the recombinant Zn- and Cd-complexes of LlwtMT, Lltr1MT, and Lltr2MT.

MT	ICP-AES ^a^	Neutral ESI-MS ^b^	Experimental MM ^c^	Theoretical MM ^d^
LlwtMT	9.5	Zn_9_-MT	10,753	10,754.1
Lltr1MT	5.9	Zn_6_-MT	6807	6806.7
Lltr2MT	6.1	Zn_6_-MT	7226	7227.2
LlwtMT	8.7	Cd_9_-MT	11,177	11,177.3
Lltr1MT	6.3	Cd_6_-MT	7089	7088.8
Lltr2MT	6.5	Cd_6_-MT	7509	7509.3

^a^ M(II)-to-peptide ratio calculated from S and Zn or Cd content (Inductively Coupled Plasma Atomic Emission Spectroscopy, ICP-AES, data); ^b^ the metal contents of the M(II)-LlMT species were calculated from the mass difference between the holo- and the respective apo-peptides; ^c^ experimental molecular masses corresponding to the detected M(II)-LlMT complexes. The corresponding ESI-MS spectra are shown in Figure 3; ^d^ theoretical molecular masses corresponding to the M(II)-LlMT complexes.

**Table 2:** Analytical characterization of the recombinant Cu-complexes of LlwtMT, Lltr1MT, and Lltr2MT, obtained from cultures grown with normal aeration (no complexes could be recovered from low aeration conditions).

MT	ICP-AES ^a^	Neutral ESI-MS ^b^	Exp MM ^c^	Theor MM ^d^	Acidic ESI-MS ^b^	Exp MM ^c^	Theor MM ^d^
LlwtMT	4.0 Zn 13.3 Cu	M_14_-MT M_13_-MT M_12_-MT M_15_-MT	11,058 10,995 10,934 11,122	11,059.3 10,996.7 10,934.2 11,121.8	Cu_12_-MT	10,934	10,934.2
					Cu_8_-MT	10,682	10,684.0
					Cu_11_-MT	10,869	10,871.6
					Cu_10_-MT	10,808	10,809.1
					Cu_13_-MT	10,995	10,996.7
					Cu_9_-MT	10,747	10,746.5
					Cu_14_-MT	11,061	11,059.3
Lltr1MT	3.5 Zn 5.8 Cu	M_8_-MT M_9_-MT M_10_-MT M_7_-MT M_6_-MT	6925 6988 7050 6862 6796	6926.8 6989.3 7051.9 6864.2 6801.7	Cu_4_-MT	6675	6676.6
					apo-MT	6425	6426.4
					Cu_8_-MT	6925	6926.8
					Cu_5_-MT	6738	6739.1
					Cu_6_-MT	6801	6801.7
					Cu_7_-MT	6865	6864.2
Lltr2MT	3.2 Zn 6.5 Cu	M_8_-MT M_9_-MT M_10_-MT M_7_-MT M_6_-MT	7344 7407 7471 7285 7224	7347.2 7409.8 7472.3 7284.7 7222.1	Cu_4_-MT	7095	7097.0
					apo-MT	6845	6846.8
					Cu_8_-MT	7344	7347.2
					Cu_5_-MT	7161	7159.6
					Cu_6_-MT	7220	7222.1
					Cu_7_-MT	7286	7284.7

^a^ The Zn(II) and Cu(I)-to-peptide ratio calculated from S and Cu content (ICP-AES data); ^b^ the deduced M-LlMT (M = Zn or Cu) species were calculated from the mass difference between the holo- and the respective apo-peptides. The major species are indicated in bold, and the rest are in decreasing order of ESI-MS peak intensity; ^c^ experimental molecular masses corresponding to the detected M-LlMT complexes. The corresponding ESI-MS spectra are shown in Figure 5; ^d^ theoretical molecular masses corresponding to the M-LlMT complexes.

## Supplementary Materials

Supplementary materials can be found at www.mdpi.com/1422-0067/18/7/1452/s1.
