# Supplementary material for: Analysis of Metal-Binding Features of the Wild Type and Two Domain-Truncated Mutant Variants of Littorina littorea Metallothionein Reveals Its Cd-Specific Character
Source: Int J Mol Sci. 2017 Jul 6;18(7):1452. doi: 10.3390/ijms18071452 (PMC5535943; doi:10.3390/ijms18071452)
Supplement: Supplementary file 1 [file ijms-18-01452-s001.pdf]

LlwtMT: wild type *Littorina littorea* MT

LlwtMT cDNA sequence for cloning in pGEX-4T-1 plasmid

TTTATTGGATCCATGAGCTCTGTGTTCGGAGCAGGTTGCACTGATGTGTGTAAGCAAACCCCATGC
GGGTGTGCAACCAGTGGATGTAACTGTACCGACGATTGCAAATGTCAGTCGTGTAAATACGGCGCC
GGGTGCACCGATACCTGCAAGCAGACGCCGTGTGGATGCGGTTCTGGGTGCAACTGCAAGGAAGAT
TGCCGCTGCCAATCGTGTTCTACGGCCTGTAAATGCGCAGCGGGCTCTTGCAAATGCGGTAAAGGT
TGTACCGGGCCTGATTCGTGTAAGTGTGACCGCAGCTGCTCATGCAAATGACTCGAGAAA

LlwtMT protein sequence

MSSVFGAGCTDVCKQTPCGCATSGCNCTDDCKCQSCKYGAGCTDTCKQTPCGCGSGCNCKEDCRCQSCSTAC
KCAAGSCKCGKGCTGPDSCKCDRSCSCK*

Lltr1MT: truncated 1 of *Littorina littorea* MT

Lltr1MT cDNA sequence for cloning in pGEX plasmid

TTTATTGGATCCATGGGTGCGGGCTGCACTGACACTTGTAAACAAACCCCATGCGGTTGCGGCAGT
GGCTGCAATTGCAAAGAAGATTGCCGTTGCCAGTCGTGTTCTACGGCATGTAAATGCGCCGCAGGT
TCTTGCAAATGCGGTAAAGGCTGCACGGGTCCAGATAGCTGTAAATGTGACCGTTCGTGCAGCTGT
AAATGACTCGAGAAA

Lltr1MT protein sequence

MGAGCTDTCKQTPCGCGSGCNCKEDCRCQSCSTACKCAAGSCKCGKGCTGPDSCKCDRSCSCK*

Lltr2MT: truncated 2 of *Littorina littorea* MT (with SSVF peptide)

Lltr2MT cDNA sequence for cloning in pGEX plasmid

TTTATTGGATCCATGAGTAGTGTATTCGGGGCAGGTTGTACGGATACCTGCAAGCAGACCCCGTGT
GGCTGCGGTAGTGGATGCAACTGCAAAGAAGATTGTCGTTGCCAGTCGTGTTCAACCGCTTGCAAA
TGCGCCGCAGGTAGCTGCAAATGCGGCAAAGGCTGCACCGGACCGGACTCTTGTAAGTGTGACCGT
TCTTGTTCATGTAAGTAACTCGAGAAA

Lltr2MT protein sequence

MSSVFGAGCTDTCKQTPCGCGSGCNCKEDCRCQSCSTACKCAAGSCKCGKGCTGPDSCKCDRSCSCK*

Figure S1. cDNA and protein sequence information of the proteins studied in this work. The constructs Lltr1MT and Lltr2MTare shown in comparison to LtwtMT. Both cDNA and protein sequences are shown, and the grey boxes indicate the 5′ and 3′ restriction sites (*BamH*I and *Xho*I, respectively) used for cloning into the pGEX-4T-1 plasmid. Highlighted in yellow is the SSVF N-term sequence and in green the first metal binding domain that is removed in the mutated proteins.
